# Impact of a national guideline for the management of peripheral arterial disease on revascularization rates in England: interrupted time series analysis

**DOI:** 10.1093/bjsopen/zrae115

**Published:** 2024-10-22

**Authors:** Ravi Maheswaran, Thaison Tong, Jonathan Michaels, Paul Brindley, Stephen Walters, Shah Nawaz

**Affiliations:** Epidemiology and Public Health, School of Medicine and Population Health, University of Sheffield, Sheffield, UK; School of Medicine and Population Health, University of Sheffield, Sheffield, UK; Clinical Decision Science, School of Medicine and Population Health, University of Sheffield, Sheffield, UK; Landscape Planning, Department of Landscape Architecture, University of Sheffield, Sheffield, UK; Medical Statistics and Clinical Trials, School of Medicine and Population Health, University of Sheffield, Sheffield, UK; Sheffield Vascular Institute, Sheffield Teaching Hospitals NHS Foundation Trust, Sheffield, UK

## Abstract

**Background:**

A national guideline on peripheral arterial disease management in England was issued in August 2012. The impact on revascularization rates was examined and variation with socioeconomic deprivation assessed.

**Methods:**

Annual hospital admissions for England over 10 years (2008–2009 to 2017–2018) were examined using interrupted time series analysis. A pragmatic approach was used to classify admissions for revascularization into moderate and severe categories.

**Results:**

There were 309 839 admissions (56% for moderate peripheral arterial disease), with an overall annual admission rate for revascularization of 86 per 100 000 population aged 25+ years. The rate for moderate peripheral arterial disease marginally increased by 0.29 per 100 000 per year (95% c.i. −0.22 to 0.80) from 2008–2009 to 2012–2013. Following guideline introduction, this rate decreased. The equivalent for severe peripheral arterial disease increased by 1.33 per 100 000 (0.78 to 1.88). Following guideline introduction, this rate plateaued. The change in rate (slope) for moderate peripheral arterial disease of −2.81 per 100 000 per year (−3.52 to −2.10) after guideline introduction was greater than the change in rate for severe peripheral arterial disease of −1.95 per 100 000 per year (−2.73 to −1.17). For moderate peripheral arterial disease, the annual rate in the most socioeconomically deprived category was 15.6 per 100 000 lower in 2017–2018 compared with 2012–2013 (24.3% decrease). The impact progressively diminished with decreasing deprivation. In the least deprived category, the reduction was 5.2 per 100 000 (12.9% decrease). For severe peripheral arterial disease, the decrease was 1.2 per 100 000 (3.1% reduction) with no consistent variation in relation to deprivation.

**Conclusion:**

Introduction of the national peripheral arterial disease management guideline in England was associated with a reduction in admission rates for revascularization, especially for moderate peripheral arterial disease, with greater reduction in rates for moderate peripheral arterial disease in more socioeconomically deprived areas. Association, however, does not necessarily imply causation and alternative explanations cannot be ruled out.

## Teaser

A national guideline for managing peripheral arterial disease was released for the English National Health Service in August 2012. The impact on revascularization rates was examined, and variation in impact associated with socioeconomic deprivation was evaluated. Introduction of the national peripheral arterial disease management guideline was associated with a reduction in admission rates for revascularization, especially for moderate peripheral arterial disease, with greater reduction in rates for moderate peripheral arterial disease in more socioeconomically deprived areas.

## Introduction

Peripheral arterial disease (PAD) affecting the lower limbs can cause symptoms ranging from intermittent claudication to limb-threatening ischaemia, which includes ischaemic rest pain, ulceration and gangrene, that could lead to amputation. The global prevalence of PAD increases with age, rising from 4.3% in low- and middle-income countries and 3.5% in high-income countries at 40–44 years, to 12.0% and 21.2% respectively at 80–84 years^1^. Treatment options for PAD range from medical and lifestyle management to surgery, depending on the severity of the condition^2,3^. Medical and lifestyle management includes smoking cessation, exercise and medication. Surgical options include a range of endovascular procedures and open surgery to carry out endarterectomy or bypass.

In England, the National Health Service (NHS) provides universal access to healthcare that is centrally funded mainly through general taxation. The National Institute for Health and Care Excellence (NICE) is the national body set up to issue standard guidelines with evidence-based recommendations for healthcare provided by the NHS in England. In 2010, NICE identified the need for a national guideline for the management of PAD^4^. The reasons for this included increasing evidence for the effectiveness of medical and lifestyle management including supervised exercise programmes but access to these programmes was variable. In addition, there was increasing use of endovascular surgery, including several new techniques, but there was uncertainty regarding benefits with wide variations in practice.

The NICE guideline on the diagnosis and management of PAD was published in August 2012, accompanied by a comprehensive review of the evidence supporting the recommendations in this guideline^5,6^. For intermittent claudication, the key recommendation was for medical and lifestyle management, with a supervised exercise programme offered to all people with this condition. With regard to angioplasty, the guidance made it clear that this was to be offered to people with intermittent claudication only when advice on the benefits of modifying risk factors had been reinforced, a supervised exercise programme had not led to a satisfactory improvement in symptoms and imaging had confirmed that angioplasty would be suitable for the person. In addition, bypass surgery for treating people with severe lifestyle-limiting intermittent claudication was only to be offered when angioplasty had been unsuccessful or unsuitable and imaging had confirmed that bypass surgery was appropriate for the person.

For chronic limb-threatening ischaemia (CLTI), the NICE guideline recommendation was for all people to be assessed by a multidisciplinary team before treatment decisions were made. The recommendation included offering angioplasty or bypass surgery to people with CLTI who required revascularization, taking into account factors such as co-morbidity and patient preference.

This study assessed the impact of this guideline on trends in surgery rates for PAD before and after this national guideline was issued. There is a socioeconomic gradient in the prevalence of PAD with higher rates in more socioeconomically disadvantaged sections of the population^7,8^. The study also assessed whether the impact varied with socioeconomic deprivation.

## Methods

### Hospital Episode Statistics

The study used Hospital Episode Statistics (HES) data for admitted patient care, including data on day case admissions, obtained from NHS England. The admission records did not contain diagnosis codes that differentiated between intermittent claudication and CLTI. A pragmatic approach was therefore used to classify PAD surgery into two categories, ‘moderate’ and ‘severe’.

The classification was established based on definitions developed in a previous vascular research programme funded by the National Institute for Health and Care Research (NIHR). This involved an iterative process wherein a clinical consensus group reviewed sample sets of full HES coding for individual cases, assigning them to categories of severe and less severe leg ischaemia. The process produced categories that demonstrated clear differences in demographic, process and outcome measures that were consistent with distinct groups based upon severity of PAD^9^.

All PAD surgery that was carried out in an emergency (unplanned) admission was classified as ‘severe’. In addition, any elective (planned) PAD surgery admission that included an ICD-10 code for leg ulcer, or that included aortoiliac bypass, femoro-distal bypass or extra-anatomical bypass was classified as ‘severe’.

The remaining elective admissions for PAD surgery were classified as surgery for ‘moderate’ PAD. The main operations in this category were femoral angioplasty, iliac angioplasty, femoral endarterectomy and femoro-proximal bypass.

If there was more than one revascularization procedure during the same admission, the admission was classified on the basis of the most ‘severe’ procedure in that admission using the previously established classification^9^. Data were analysed for the 10 years, 2008–2009 to 2017–2018 (data are provided for financial years which run from 1 April to 31 March of the following year). This time span allowed for analysis of a similar number of years before and after publication of the PAD guideline.

### Study design

The study design employed to assess the impact of the guideline on population-based surgery rates for PAD was a ‘before and after’ study design. It was implemented using an interrupted time series analysis. The impact of the guideline was assessed separately for revascularization for moderate and severe disease. The analysis also examined if any impact of the guideline differentially affected rates in areas with different levels of socioeconomic deprivation.

### Geography, population data and socioeconomic deprivation

Each admission in the HES data set had been assigned to a lower layer super output area (LSOA) of residence. These are census areas with an average population of 1500 people^10^. As there were a small number of changes to LSOAs between the 2001 and 2011 national population censuses, the analysis was restricted to 31 672 of the 32 844 LSOAs in 2011 (96.4%) to maintain consistency across the study time span. LSOA mid-year population estimates from 2008 to 2017 were used to calculate annual admission rates for revascularization. The Income Domain from the Index of Multiple Deprivation (IMD) 2010 was used as the indicator of socioeconomic deprivation at the LSOA level^11^. The IMD is the national index of deprivation widely used by government agencies in England.

### Statistical analysis

Interrupted time series analysis was carried out using segmented regression. The outcome variable was the annual admission rate (from 2008–2009 to 2017–2018) for revascularization per 100 000 population aged 25+ years. Separate models were implemented for surgery for moderate and severe PAD. The main analysis used linear regression, with a Poisson model also employed as a sensitivity analysis. The model estimated three coefficients: the admission rate for revascularization over time (the slope of the line) before the intervention (publication of the guideline), the change in the level of the admission rate for revascularization after the intervention and the change in the slope of the line after the intervention.

As publication of the guideline in August 2012 was part-way through 2012–2013, this HES year was assigned to the preintervention interval. There were, therefore, 5 years in the preintervention interval (2008–2009 to 2012–2013) and 5 years in the postintervention interval (2013–2014 to 2017–2018). The main set of analyses were carried out on this basis.

## Results

There was a total of 309 839 admissions over the 10-year study interval, with a corresponding population aged 25+ years of 36 million, giving an overall annual admission rate for PAD-related admissions for revascularization of 86 per 100 000 population; 56% of revascularization admissions were for moderate PAD and the remaining 44% were for severe PAD.

Patients admitted for revascularization for moderate PAD were marginally younger, with a higher proportion of men, than admissions for revascularization for severe PAD (*Table 1*). The great majority of admissions in both categories (>90%) were of White ethnicity. Angioplasty accounted for 79.4% of moderate PAD admissions (80.1% pre- and 78.6% postintervention), and 54% of severe PAD admissions (51.5% pre- and 56.3% postintervention). Characteristics of admissions in the 5-year interval before and the 5-year interval after the guideline were broadly similar (*Table 1*).

**Table 1:** Characteristics of admissions for revascularization for PAD; England (1 April 2008–31 March 2018)

Characteristics	Preintervention interval (5 years from 1 April 2008 to 31 March 2013)	Postintervention interval (5 years from 1 April 2013 to 31 March 2018)	All years
**Revascularization for moderate PAD**			
N	89 825	84 189	174 014
Age (years), mean(s.d.)	68.4(10.9)	68.5(11.0)	68.4(10.9)
Men (%)	69.2	69.2	69.2
Deprivation category			
1 (most deprived)	20 529 (22.9)	19 176 (22.8)	39 705 (22.8)
2	19 462 (21.7)	18 288 (21.7)	37 750 (21.7)
3	18 625 (20.7)	17 131 (20.3)	35 756 (20.5)
4	16 879 (18.8)	15 941 (18.9)	32 820 (18.9)
5 (least deprived)	14 330 (16.0)	13 653 (16.2)	27 983 (16.1)
Total	89 825 (100)	84 189 (100)	174 014 (100)
Ethnicity			
White	85 427 (95.1)	78 616 (93.4)	164 043 (94.3)
Black	920 (1.0)	901 (1.1)	1821 (1.0)
Asian	1459 (1.6)	1511 (1.8)	2970 (1.7)
Mixed	209 (0.2)	226 (0.3)	435 (0.2)
Others	542 (0.6)	629 (0.7)	1171 (0.7)
Missing	1268 (1.4)	2306 (2.7)	3574 (2.1)
Total	89 825 (100)	84 189 (100)	174 014 (100)
Operation type			
(All were elective admissions with no leg ulcers)			
Femoro-proximal bypass	9571 (10.7)	8145 (9.7)	17 716 (10.2)
Femoral endarterectomy	8300 (9.2)	9845 (11.7)	18 145 (10.4)
Iliac angioplasty	22 864 (25.5)	20 787 (24.7)	43 651 (25.1)
Femoral angioplasty	32 637 (36.3)	33 613 (39.9)	66 250 (38.1)
Unspecified angioplasty	16 453 (18.3)	11 799 (14.0)	28 252 (16.2)
Total	89 825 (100)	84 189 (100)	174 014 (100)
**Revascularization for severe PAD**			
N	64 132	71 693	135 825
Age (years), mean(s.d.)	70.8(12.9)	70.5(13.0)	70.6(12.9)
Men (%)	62.8	64.8	63.8
Deprivation category			
1 (most deprived)	15 556 (24.3)	17 436 (24.3)	32 992 (24.3)
2	13 967 (21.8)	16 036 (22.4)	30 003 (22.1)
3	13 324 (20.8)	14 739 (20.6)	28 063 (20.7)
4	11 802 (18.4)	12 910 (18.0)	24 712 (18.2)
5 (least deprived)	9483 (14.8)	10 572 (14.7)	20 055 (14.8)
Total	64 132 (100)	71 693 (100)	135 825 (100)
Ethnicity			
White	60 507 (94.3)	66 393 (92.6)	126 900 (93.4)
Black	1178 (1.8)	1507 (2.1)	2685 (2.0)
Asian	1100 (1.7)	1601 (2.2)	2701 (2.0)
Mixed	171 (0.3)	227 (0.3)	398 (0.3)
Others	476 (0.7)	712 (1.0)	1188 (0.9)
Missing	700 (1.1)	1253 (1.7)	1953 (1.4)
Total	64 132 (100)	71 693 (100)	135 825 (100)
Operation type			
(Elective and emergency admissions)			
Aortoiliac bypass	7845 (12.2)	7416 (10.3)	15 261 (11.2)
Femoro-distal bypass	4366 (6.8)	4728 (6.6)	9094 (6.7)
Extra-anatomical bypass	5317 (8.3)	4078 (5.7)	9395 (6.9)
(Emergency admissions or elective admissions with leg ulcers)			
Femoro-proximal bypass	8411 (13.1)	8652 (12.1)	17 063 (12.6)
Femoral endarterectomy	5160 (8.0)	6485 (9.0)	11 645 (8.6)
Iliac angioplasty	5569 (8.7)	6255 (8.7)	11 824 (8.7)
Femoral angioplasty	18 479 (28.8)	24 214 (33.8)	42 693 (31.4)
Unspecified angioplasty	8985 (14.0)	9865 (13.8)	18 850 (13.9)
Total	64 132 (100)	71 693 (100)	135 825 (100)

Values are *n* (%) unless otherwise indicated. PAD, peripheral arterial disease.

**Fig. 1:**
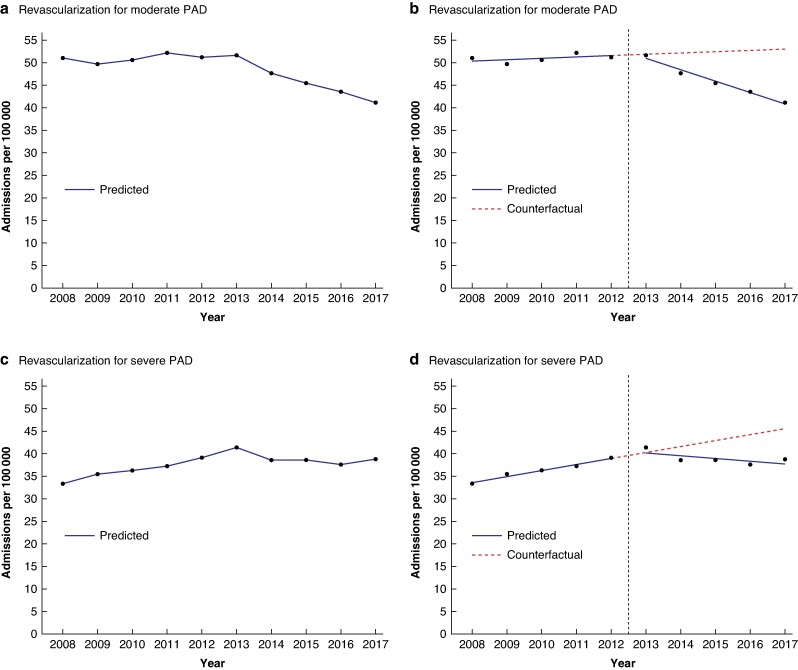
Observed time trends in admission rates (per 100 000 population per year) for revascularization for moderate and severe PAD, accompanied by modelled linear trends from the interrupted time series analysis; England (1 April 2008–31 March 2018)

The annual admission rate for revascularization for moderate PAD marginally increased in the interval before the guideline was introduced (*Fig. 1*) by 0.29 per 100 000 population per year (95% c.i. −0.22 to 0.80) (*Table 2*). Following introduction of the guideline, this admission rate decreased.

PAD, peripheral arterial disease.

**Table 2:** Results of the interrupted time series analysis of the impact of the PAD guideline on annual admission rates for revascularization for PAD; England (1 April 2008–31 March 2018)

	Estimate (admissions per 100 000 population per year)	95% c.i.	*P*
**Revascularization for moderate PAD**			
Intercept (admission rate in 2008–2009)	50.05	(48.38,51.72)	<0.001
Trend before the guideline (annual increase in admission rate from 2008–2009 to 2012–2013)	0.29	(−0.22,0.80)	0.30
Level change after the guideline (step change in rate in 2013–2014)	1.90	(−0.16,3.96)	0.12
Change in trend after the guideline (annual change in trend from 2013–2014 to 2017–2018)	−2.81	(−3.52,−2.10)	<0.001
Change in admission rate: 2017–2018 compared with 2012–2013 (calculated using above results)			
Absolute change (admissions per 100 000 population per year)	−10.7		
Relative change (%)	−20.8%		
**Revascularization for severe PAD**			
Intercept (admission rate in 2008–2009)	32.36	(30.52,34.20)	< 0.001
Trend before the guideline (annual increase in admission rate from 2008–2009 to 2012–2013)	1.33	(0.78,1.88)	<0.001
Level change after the guideline (step change in rate in 2013–2014)	1.89	(−0.38,4.16)	0.16
Change in trend after the guideline (annual change in trend from 2013–2014 to 2017–2018)	−1.95	(−2.73,−1.17)	<0.001
Change in admission rate: 2017–2018 compared with 2012–2013 (calculated using above results)			
Absolute change (admissions per 100 000 population per year)	−1.2		
Relative change (%)	−3.1%		

PAD, peripheral arterial disease.

The admission rate for revascularization for severe PAD increased by 1.33 per 100 000 population per year (95% c.i. 0.78 to 1.88) before publication of the guideline. Following the introduction of the guideline, this rate essentially plateaued (*Fig. 1*, *Table 2*).

The change in trend in the admission rate for revascularization for moderate PAD of −2.81 per 100 000 population per year (95% c.i. −3.52 to −2.10) after introduction of the guideline was greater than the change in trend in the admission rate for revascularization for severe PAD of −1.95 per 100 000 per year (95% c.i. −2.73 to −1.17) (*Table 2*).

Results from the interrupted time series analysis were used to compare rates in 2017–2018 with those in 2012–2013 (the last year assigned to the preintervention interval) in absolute and relative terms. For revascularization for moderate PAD, the admission rate in 2017–2018 had decreased by 10.7 per 100 000 population compared with the rate in 2012–2013. In relative terms, this was a 20.8% reduction in the annual rate in 2017–2018 compared with 2012–2013 (*Table 2*).

For revascularization for severe PAD, the admission rate in 2017–2018 had decreased by 1.2 per 100 000 population compared with the rate in 2012–2013. In relative terms, this was a 3.1% reduction in the rate in 2017–2018 compared with 2012–2013 (*Table 2*).

As the rate for moderate PAD appeared to continue to increase for a further year (2013–2014) following the year in which the guideline was published (*Fig. 1*), this could have represented a lag interval before full implementation of the guideline. A sensitivity analysis was therefore carried out, allocating 6 years (2008–2009 to 2013­–2014) to the preintervention interval and 4 years (2014–2015 to 2017) to the postintervention interval in the interrupted time series analysis. Sensitivity analyses were also carried out omitting the level change variable in the interrupted time series analysis because this variable was not significant, and it could be argued that a step change would have been unlikely as implementation of the PAD guideline would have been relatively gradual. In addition, sensitivity analysis was carried using Poisson instead of linear regression as admissions are count data. All sensitivity analyses, however, produced results that were broadly consistent with the results above from the main analysis.

### Impact of the guideline in relation to socioeconomic deprivation

**Fig. 2:**
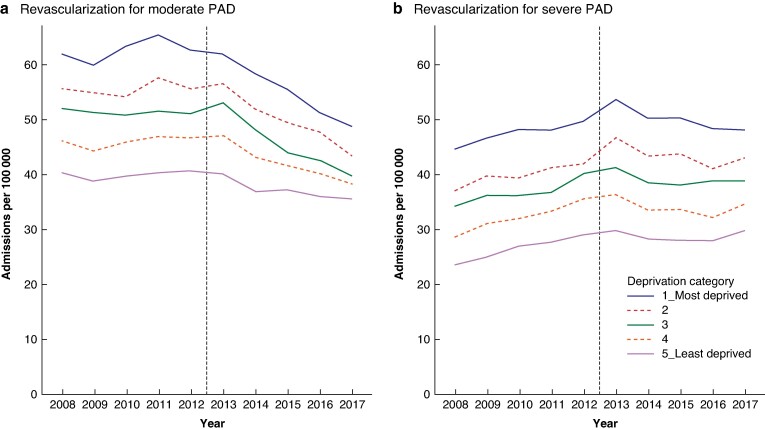
Socioeconomic variation in time trends in admission rates (per 100 000 population per year) for revascularization for moderate and severe PAD before and after publication of the PAD guideline; England (1 April 2008–31 March 2018)

Admission rates for revascularization were progressively higher with higher levels of socioeconomic deprivation, and this was the case for both moderate and severe PAD (*Fig. 2*).

PAD, peripheral arterial disease.

With regard to the impact of the guideline on admission rates for revascularization by deprivation quintile category, the greatest impact for moderate PAD was seen in the most deprived category in both absolute and relative terms (*Table 3*). The admission rate for revascularization for moderate PAD decreased by 15.6 per 100 000 population in the most deprived category in absolute terms between 2012–2013 and 2017–2018. In relative terms, this was a 24.3% decrease. The magnitude of the impact on moderate PAD progressively diminished with decreasing levels of deprivation. In the least deprived category, the absolute reduction in the rate in 2017–2018 compared with 2012–2013 was 5.2 per 100 000 population, a 12.9% decrease in relative terms.

**Table 3:** Results of the interrupted time series analysis, by socioeconomic deprivation category, of the impact of the PAD guideline on annual admission rates (per 100 000 population) for revascularization for PAD; England (1 April 2008–31 March 2018)

	Socioeconomic deprivation category					
	1 (most deprived)	2	3	4	5 (least deprived)	All
**Revascularization for moderate PAD**						
Intercept	60.60	54.81	51.84	44.87	39.55	50.05
Trend before the guideline	0.70	0.28	−0.15	0.39	0.17	0.29
Level change after the guideline	1.17	2.80	4.09	1.48	−0.17	1.90
Trend change after the guideline	−4.05	−3.36	−3.06	−2.44	−1.17	−2.81
Change in admission rate: 2017–2018 compared with 2012–2013 (calculated using above results)						
Absolute change (admissions per 100 000 per year)	−15.6	−12.6	−12.0	−8.8	−5.2	−10.7
Relative change (%)	−24.3%	−22.3%	−23.4%	−18.8%	−12.9%	−20.8%
**Revascularization for severe PAD**						
Intercept	44.00	36.55	33.06	27.29	22.39	32.36
Trend before the guideline	1.16	1.12	1.23	1.63	1.37	1.33
Level change after the guideline	4.26	4.35	1.29	0.11	−0.33	1.89
Trend change after the guideline	−2.47	−2.08	−1.67	−2.10	−1.40	−1.95
Change in admission rate: 2017–2018 compared with 2012–2013 (calculated using above results)						
Absolute change (admissions per 100 000 per year)	−2.3	−0.4	−0.9	−2.3	−0.5	−1.2
Relative change (%)	−4.6%	−1.1%	−2.4%	−6.4%	−1.7%	−3.1%

PAD, peripheral arterial disease.

In contrast, with regard to the admission rate for revascularization for severe PAD, there was no evidence of consistent variation in impact in relation to socioeconomic deprivation, either in absolute or in relative terms (*Table 3*).

## Discussion

This study found that the NICE guideline on diagnosis and management of PAD was associated with a greater impact on admission rates for revascularization for moderate than for severe PAD. The decrease in admission rates for revascularization for moderate PAD following introduction of the guideline was greater in both absolute and relative terms than the decrease for severe PAD. The impact on moderate PAD progressively increased with increasing socioeconomic deprivation, with the greatest reductions in admission rates for revascularization seen in the most deprived category in both absolute and relative terms. In contrast, the admission rates for revascularization for severe PAD showed no evidence of a consistent association with deprivation.

This is the first study to have examined the impact of a national PAD guideline on population-based admission rates for revascularization for PAD. A previous study reported on adherence to guideline-recommended revascularization in a cohort of patients in Germany and found that adherence was generally inadequate in patients with CLTI^12^. Previous studies have also examined adherence to guideline-recommended medical management of PAD in several countries, including the UK, USA, Germany, Netherlands and Canada, and generally found suboptimal use of recommended medications^13–18^.

The interrupted time series study design is a useful quasi-experimental design for evaluating longitudinal effects of interventions and causal inference from observational data^19^. However, the association observed may not have been causally linked as other factors, such as organizational changes in vascular surgery, wider changes in governmental health policy, funding constraints and austerity measures, coincidentally occurred broadly around the time the PAD guideline was issued. If these factors were the cause of the sharp change in rates observed, however, it might be expected that a similar pattern in surgery rates for other vascular conditions might also have occurred.

With regard to surgery for carotid artery disease, there were no similar changes in rates around 2012–2013^20^. Elective open repair rates for abdominal aortic aneurysm continuously decreased between 2006–2007 and 2017–2018 while elective endovascular aneurysm repair rates increased to a peak in 2011–2012 and started decreasing from 2012–2013 onwards^21^, whereas PAD admission rates for revascularization observed in the present study had not decreased yet in 2012–2013. Varicose vein surgery rates had a U-shaped decrease and increase, starting in 2010–2011 and recovering to around previous levels by 2014–2015^22^. Major lower limb amputation rates generally decreased over time except for men with diabetes for whom rates plateaued from 2011–2012 onwards^23^. Over the interval of the study there was a significant change in the delivery of vascular services, with subspecialization, a reduction in the number of centres offering such services and moves towards a hub and spoke arrangement. However, this was a gradual process over the full interval of the study. Overall, therefore, there is little to suggest that organizational changes in vascular surgery or funding constraints could have accounted for the sharp change in rates following issue of the guideline observed in the present study.

It may be the case that the NICE guidance is not causally related to changes in practice, but that guidance reflects altered clinical approaches or responses to emerging evidence that are, themselves, driving such changes. However, considerable resources are devoted to the development and dissemination of NICE guidance with the intention of influencing clinical practice, and the purpose of the present study was to consider whether there is evidence that would support a potential effect of NICE guidance on clinical practice and, if so, whether the extent of such effects was linked to socioeconomic factors.

Smoking and diabetes are important risk factors for PAD. Although there is little information specifically on smoking trends in patients with PAD over the 10-year time interval examined, smoking prevalence in England gradually decreased over this time interval^24^, while diabetes prevalence gradually increased^25^. The present study found that socioeconomic inequalities in revascularization for moderate PAD narrowed after the NICE guideline was issued, in contrast to inequalities in health in general in England, which widened over the 10-year time interval examined^26,27^. These wider trends, therefore, are unlikely to have explained the sharp change in rates following issue of the guideline.

If the NICE guideline did result in a reduction in revascularization rates for moderate PAD, the question that arises is the mechanism by which this could have occurred. It is unlikely to have been through supervised exercise programmes as there is evidence that these were inadequately implemented^28,29^. In addition, rates of guideline-recommended therapy for PAD were well below expected levels^18^. A potential explanation is that in the resource-constrained NHS environment, the guideline could have given hospital surgical management the opportunity to cut back on surgery for moderate PAD to manage costs and pressures on hospital beds and waiting lists, even if other recommendations in the guideline were not being adequately implemented.

Implementation of evidence-based guidelines in surgical practice may be delayed or not implemented^30,31^, but changes in practice can sometimes also anticipate emerging evidence^31^. Trends in vascular surgical practice towards focusing more on revascularization for CLTI and reducing surgical intervention for intermittent claudication may have contributed to the trends observed in revascularization rates in the present study. However, the sharp change in rates following issue of the guideline suggests that the guideline may well have been the key contributor to the pattern seen.

The observed reduction in admission rates for revascularization for severe PAD was unexpected as the guideline did not recommend any reduction in revascularization for patients with CLTI. One possible explanation for this apparent anomaly is that the pragmatic approach used to define surgery for PAD may have inadvertently included some patients with intermittent claudication in the severe category. The observed reduction in the admission rate for revascularization in this category could therefore be explained by appropriate reduction in the use of this procedure for intermittent claudication. Another potential explanation is that the demarcation between intermittent claudication and CLTI is not rigid, and maximizing non-surgical management instead of revascularization may have been appropriate for some severe PAD patients. A third potential explanation is that possible misinterpretation of the guideline may have inadvertently resulted in revascularization not being considered in some patients with severe PAD.

The higher rates of revascularization observed in this study in more socioeconomically deprived areas is consistent with the higher prevalence of PAD in more disadvantaged groups reported in previous studies^7,8^. For example, a record linkage study in England calculated that cumulative lifetime incidence of PAD in men was 7.8% in the most deprived areas by quintile, compared with 4.6% in the least deprived areas^7^. The corresponding figures for women were 5.8% and 4.3% respectively. In the USA, the prevalence of PAD was more than twice as high in the most disadvantaged of six categories of socioeconomic disadvantage compared with the least disadvantaged category^8^.

The greater impact of the guideline in terms of reduction in admission rates for revascularization for moderate PAD in more socioeconomically deprived areas is a clear new finding from this study. This finding suggests that the guideline might have been effective in reducing socioeconomic inequalities in unnecessary revascularization for moderate PAD. This interpretation assumes that a higher proportion of avoidable revascularizations were previously being carried out on patients living in more deprived areas, possibly because effective medical and lifestyle management measures such as supervised exercise programmes had been inadequately implemented in these areas. In addition, for severe PAD, for which revascularization is a surgically appropriate intervention, the guideline did not appear to have any differential socioeconomic impact. This implies that the guideline did not worsen any socioeconomic disparities in surgical treatment for severe PAD despite the unexpected apparent reduction in admission rates for revascularization for severe PAD.

An alternative explanation for the narrowing in socioeconomic differences in revascularization rates for moderate PAD may be that patients from less disadvantaged areas were more assertive in seeking interventional treatment for less severe symptoms and less subject to guidance that might have been seen as restricting access to such services on economic grounds. Conversely, patients from more disadvantaged areas may have been less empowered to seek referral and interventional treatment for less severe symptoms, which could explain why the decrease in revascularization rates for moderate PAD was greater in more deprived areas. The assumption underlying this explanation, however, is that revascularization for moderate PAD previously being carried out before guideline implementation was mostly an appropriate procedure, which is at odds with NICE’s rationale for the guideline^5,6^. Further support for the NICE rationale comes from a recent meta-analysis of randomized clinical trials of treatment for intermittent claudication which has confirmed that non-invasive treatment is equivalent to revascularization with regard to long-term outcomes, but the latter was associated with further revascularization procedures^32^. Of concern, however, is that the greater reduction in revascularization for moderate PAD in more disadvantaged areas is unlikely to have been accompanied by an adequate increase in supervised exercise programmes. This inconsistent uptake of NICE guidance may potentially have widened socioeconomic inequalities in quality of life for people with moderate PAD.

A key strength of the HES data set is that data capture is likely to have been complete. Interrupted time series analysis using segmented regression is a useful study design for assessing causal inference in observational studies. This was a nationwide study and although there was no control group, comparison with surgery for severe PAD served as a useful comparison group to gain further insight into changes in trends.

There are, however, a number of limitations that need to be considered. There may have been errors in operative procedure coding. The HES data set is essentially an administrative data set with limited clinical information, including lack of codes to differentiate between intermittent claudication and CLTI. The pragmatic classification used instead to classify PAD surgery into categories of surgery for moderate and severe disease could have resulted in some overlap and inclusion of intermittent claudication and CLTI in both these categories. For example, some patients with CLTI may have been seen as emergencies and sent home with a planned (elective) admission within the next few days and, therefore, been misclassified in the moderate category based on mode of admission. In addition, the unit of analysis used was admissions, which meant that a patient could have had more than one admission for revascularization over the 10-year time interval examined. Also, the small number of patients from minority ethnic groups meant that ethnic variation in impact of the guideline on PAD surgery could not be usefully assessed.

A potential alternative data source for vascular surgery is the National Vascular Registry (NVR) for vascular surgery in England^33^. However, whilst estimated completeness of ascertainment of open revascularization for PAD performed in NHS hospitals was good at around 90% in the NVR, completeness of ascertainment of endovascular revascularization was much lower at around 40%^34^. Two further limitations of the NVR data are improving ascertainment over time, which makes it difficult to reliably identify time trends in PAD surgery rates over the 2008–2009 to 2017–2018 time interval, and the lack of linkage to LSOAs, which means that population-based rates by socioeconomic deprivation cannot be easily calculated. It is also worth noting that for the PAD procedures ascertained in the NVR, there was good agreement with HES in relation to procedure type (95.4% were the same) and mode of admission (elective or emergency, 91.9% were the same)^34^. This indicates that there are no major issues with the accuracy of coding of these two variables used in the pragmatic classification of PAD surgery in the present study.

This study found evidence of association between introduction of the NICE guideline on management of PAD and changes in admission rates for revascularization for PAD. There was also evidence that the guideline was associated with a greater reduction in admission rates for revascularization for moderate PAD in more socioeconomically deprived areas, and one interpretation is that the guideline might have resulted in a reduction in socioeconomic inequalities in avoidable revascularization for moderate PAD. However, association does not necessarily imply causation, and alternative explanations for the observed patterns in PAD revascularization rates which are unrelated to introduction of the PAD guideline cannot be ruled out. In addition, the guideline may not have improved overall management of PAD as key aspects of the guideline regarding supervised exercise and medical treatment had not been adequately implemented.

## Data Availability

The Hospital Episode Statistics (HES) data used in this project may be obtained from NHS England.
